# The absence of AhR in CD4^+^ T cells in patients with acute graft-versus-host disease may be related to insufficient CTCF expression

**DOI:** 10.1186/s13148-022-01330-7

**Published:** 2022-09-02

**Authors:** Cong Zeng, Ting-ting Cheng, Xia Ma, Yi Liu, Juan Hua, Xu Chen, Shi-yu Wang, Ya-jing Xu

**Affiliations:** 1 Department of Hematology, Xiangya Hospital, Central South University, Changsha, Hunan China; 2 National Clinical Research Center for Geriatric Diseases, Xiangya Hospital, Changsha, China; 3 Hunan Hematology Oncology Clinical Medical Research Center, Changsha, China; 4 National Clinical Research Center for Hematologic Diseases, The First Affiliated Hospital of Soochow University, Suzhou, China

**Keywords:** aGVHD, AhR, CTCF, TET2, EP300

## Abstract

**Background:**

Acute graft-versus-host disease (aGVHD) is a life-threatening complication of allogeneic hematopoietic stem cell transplantation (allo-HSCT). Accumulating evidence suggests that imbalanced Treg/Th17 ratio accelerates the progression of aGVHD. The aryl hydrocarbon receptor (AhR) is a basic helix-loop-helix transcription factor activated through cognate ligand binding. Current evidence supports that AhR plays a critical regulatory role in the differentiation of Treg and Th17 cells. However, the relationship between AhR and aGVHD remains unclear.

**Results:**

Our results showed that AhR expression was downregulated significantly in CD4^+^ T cells from patients with aGVHD compared with the non-aGVHD group. We also discovered that after activating AhR deficient CD4^+^ T cells, the expression levels of the activation markers-CD40L, CD134 and CD137 and cell proliferation activity were significantly higher than those of AhR-expressing CD4^+^ T cells. Restoring the expression of AhR in aGVHD CD4^+^ T cells resulted in significantly increased percentage of Tregs and associated gene transcripts, including Foxp3, IL-10 and CD39. In contrast, Th17 cell amounts and the transcription of related genes, including RORγt, IL-17A and IL-17F, were significantly reduced. We confirmed that CTCF recruited EP300 and TET2 to bind to the AhR promoter region and promoted AhR expression by mediating histone H3K9/K14 hyperacetylation and DNA demethylation in this region. The low expression of CTCF caused histone hypoacetylation and DNA hypermethylation of the AhR promoter, resulting in insufficient expression in aGVHD CD4^+^ T cells.

**Conclusions:**

CTCF is an important inducer of AhR transcription. Insufficient expression of CTCF leads to excessive AhR downregulation, resulting in substantial CD4^+^ T cell activation and Th17/Treg ratio increase, thereby mediating the occurrence of aGVHD.

## Introduction

Although allogeneic hematopoietic stem cell transplantation (allo-HSCT) is considered the exclusive curative treatment in hematopoietic malignancies, acute graft-versus-host disease (aGVHD) is a leading cause of non-relapse mortality following allo-HSCT [1–3]. aGVHD is a complex inflammatory process, in which the immune response is triggered by alloreactive donor T lymphocytes recognizing the host alloantigen. Upon activation, alloreactive donor T cells migrate to target organs, primarily the skin, lung, liver and gastrointestinal tract, where they cause tissue damage characteristic of aGVHD [4–6]. Therefore, controlling the excessive activation of T lymphocytes is the key to preventing and treating aGVHD.

The imbalanced ratio of the CD4^+^ T cell subtypes Treg and Th17 cells (Treg/Th17) accelerates the progression of aGVHD [7–9]. Tregs suppress various T cell-associated inflammatory diseases and alleviate GVHD without weakening the graft-versus-leukemia effect [10, 11]. Clinical studies have shown that Treg rate and Foxp3 expression are significantly lower in severe and mild aGVHD cases compared with patients without aGVHD and healthy donors [12, 13]. Th17 cells are relevant to the pathophysiology of aGVHD and significantly contribute to disease severity [14, 15]. Th17 cell rate and RORγt expression are significantly higher in severe and mild aGVHD cases than in patients without aGVHD and healthy donors [12]. Therefore, exploring the molecular mechanism of Treg/Th17 imbalance is the key to analyzing the pathogenesis of aGVHD and developing adequate treatment.

The aryl hydrocarbon receptor (AhR) is a basic helix-loop-helix transcription factor activated through cognate ligand binding. It is located in the cytosol of cells as an inactive complex with numerous proteins. Upon ligand binding, the AhR translocates to the nucleus and undergoes conformational changes, recruiting coactivators or corepressors to bind to the promoters of target genes and regulate their expression [16, 17]. Current evidence reveals that the AhR plays a critical role in the regulation of the immune system [18, 19], specifically the differentiation of Treg and Th17 cells [20–23]. AhR activation by 2,3,7,8-tetrachlorodibenzo-p-dioxin (TCDD), (1′H-indole-3′-carbonyl)-thiazole-4-carboxylic acid methyl ester (ITE), Kynurenine (Kyn), or Laquinimod metabolites increases Foxp3^+^ Treg cells through different mechanisms, including direct transactivation and the induction of epigenetic modifications that control Foxp3 transcription [23–27]. On the other hand, AhR activation by 6-Formylindolo [3,2-b] carbazole (FICZ) boosted Th17 cell differentiation by inducing the expression of RORC [28]. These divergent outcomes following AhR activation have been interpreted as reflecting ligand-intrinsic effects on CD4^+^ T cell differentiation, although no definitive explanation for such differences has been reported.

AhR has been confirmed to inhibit the expression of aGVHD-related inflammatory factors such as IL-1β, IL-6, and NLRP3 [29, 30]. TCDD was reported to induce regulatory function in CD4^+^ T cells suppressing murine aGVHD in an AhR-dependent mechanism [31]. In addition, studies have found that recipient mice receiving AhR^-/-^ T cells have improved survival and decreased aGVHD in two different murine allogeneic bone marrow transplant models [32]. Therefore, the relationship between AhR and aGVHD remains unknown, and the expression status of AhR in human aGVHD CD4^+^ T cells is also unclear.

In this study, we demonstrated that the AhR was downregulated in CD4^+^ T cells from patients with aGVHD compared with non-aGVHD cases. To explore the molecular mechanism of AhR downregulation in CD4^+^ T cells in aGVHD, we used bioinformatics and found that the AhR promoter region contains multiple CCCTC binding factor (CTCF) binding sites, as well as multiple CpG islands. It was reported that CTCF binding to target genes leads to DNA demethylation in the occupied region, but the specific mechanism is unclear [33]. Protein–protein interaction analysis showed that CTCF could interact with TET2 and EP300.

TET2, a member of the ten-eleven translocation (TET) family, can oxidize 5-methylcytosine (5mC) to 5-hydroxymethylcytosine (5hmc), thus promoting DNA demethylation [34]. E1A binding protein p300 (EP300) represents a histone acetyltransferase that regulates transcription via chromatin remodeling and is important in the processes of cell proliferation and differentiation [35]. Histone H3 Lys 9 (H3K9) and H3K14 are common acetylation sites for EP300 modifications and are considered hallmarks of activated gene transcription [36, 37]. Our previous studies confirmed that CTCF expression is reduced in CD4^+^ T cells from aGVHD patients compared with non-aGVHD cases [38]. Therefore, we speculated that low expression of CTCF may reduce the interaction of TET2 with EP300 in the AhR promoter and induce AhR promoter DNA hypermethylation and histone H3K9/K14 hypoacetylation, leading to insufficient AhR expression.

## Results

### AhR is decreased in CD4^+^ T cells from patients with aGVHD

**Fig. 1:**
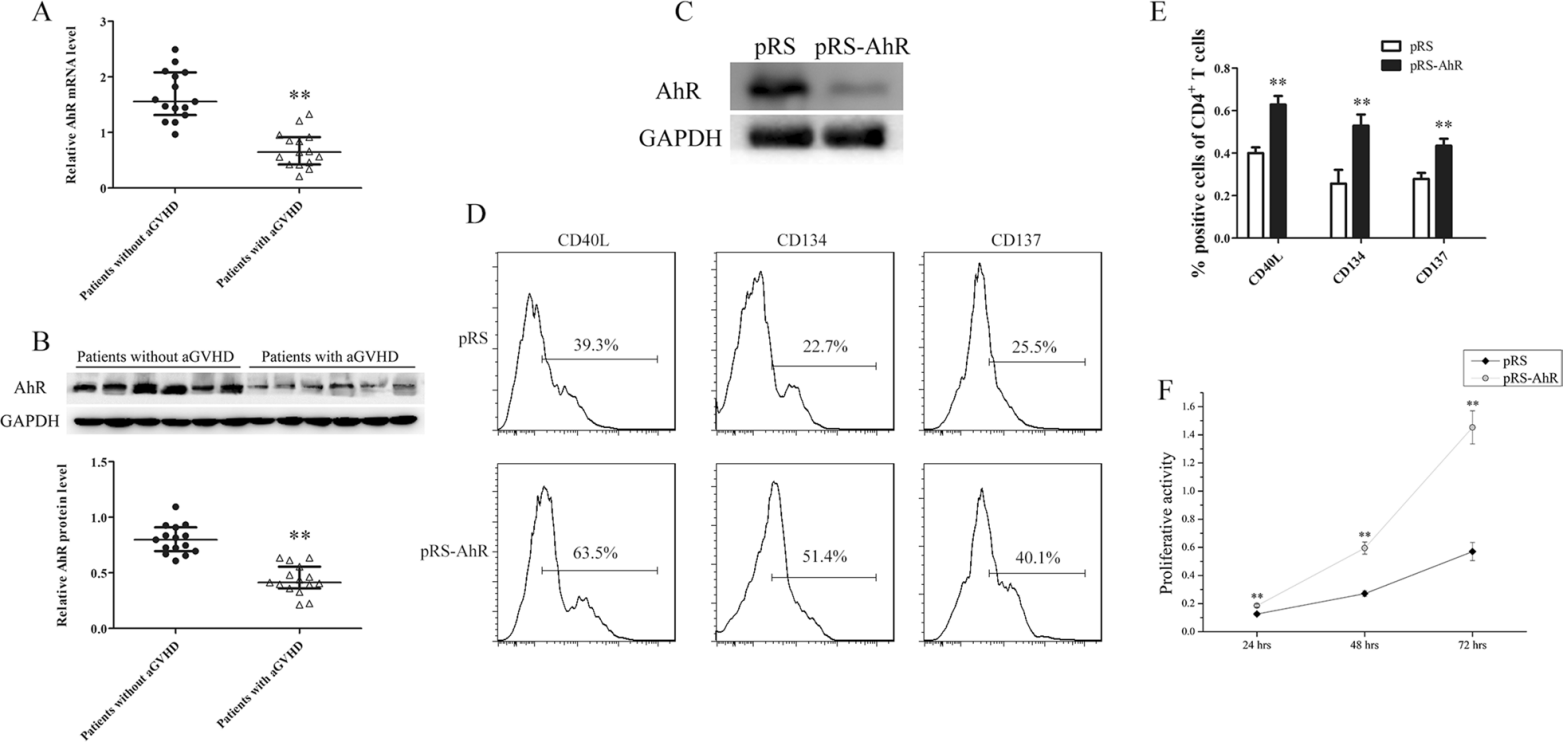
Expression of AhR in CD4^+^ T cells from patients with aGVHD and non-aGVHD cases. **A** Relative mRNA levels of AhR in CD4^+^ T cells from patients with aGVHD (*n* = 15) and non-aGVHD cases (*n* = 15), normalized to GAPDH. **B** Representative Western blot results for AhR protein expression in CD4^+^ T cells from patients with aGVHD and non-aGVHD cases (Top). Quantitative analysis of band intensities for AhR protein levels normalized by GAPDH (Bottom) (patients with aGVHD: *n* = 15, patients without aGVHD: *n* = 15). **C** Representative Western blot showing AhR protein levels in normal CD4^+^ T cells transfected with AhR interference (pRS-AhR) or negative control (pRS) plasmid. **D** The transfected cells were cultured with AhR agonist ITE and stimulated with Dynabeads T cell expander. Flow cytometry was performed to assess the expression levels of T cell activation markers (CD40L, CD134 and CD137) in different transfection groups. **E** Statistical analysis of flow cytometry data. **F** Normal CD4^+^ T cells were transfected with pRS-AhR or pRS plasmid, and treated with ITE, in the presence of anti-CD3/anti-CD28 antibodies with IL2. The CCK8 assay was performed to detect cell proliferation. Data represent the means of three independent experiments (**P < 0.01)

To investigate the AhR is abnormally expressed in CD4^+^ T cells from GVHD patients, AhR expression levels in CD4^+^ T cells from aGVHD and non-aGVHD patients were examined. Real-time PCR and Western blot showed that AhR expression was downregulated significantly in CD4^+^ T cells from patients with aGVHD compared with the non-aGVHD group (Fig. 1A, B).

To assess the effect of decreased AhR expression on CD4^+^ T cell activation, we transfected the AhR interference (pRS-AhR) or negative control (pRS) plasmid into CD4^+^ T cells from healthy donors. The transfected cells were cultured with AhR agonist ITE and subsequently stimulated with Dynabeads T cell expander (TCR stimulation). Western blot showed that AhR expression in the pRS-AhR group was significantly reduced compared with the pRS group (Fig. 1C). Flow cytometry was performed to detect the expression levels of T cell activation markers (CD40L, CD134 and CD137) in different transfection groups; compared with the pRS group, the expression levels of CD40L, CD134 and CD137 were significantly higher in the pRS-AhR group (Figures. 1D-E). In addition, to examine the effect of AhR downregulation on CD4^+^ T cell proliferation, normal CD4^+^ T cells were transfected with pRS-AhR and pRS plasmids, respectively, and administered ITE in the presence of anti-CD3/anti-CD28 antibodies with IL2. The CCK8 assay showed that the proliferation of cells transfected with pRS-AhR was significantly higher than that of the pRS group (Fig. 1F). These results indicated that AhR downregulation may be an important reason for the overactivation and proliferation of aGVHD CD4^+^ T cells.

### Restoring AhR expression in aGVHD CD4^+^ T cells increases Treg cell amounts and reduces Th17 cell levels

**Fig. 2:**
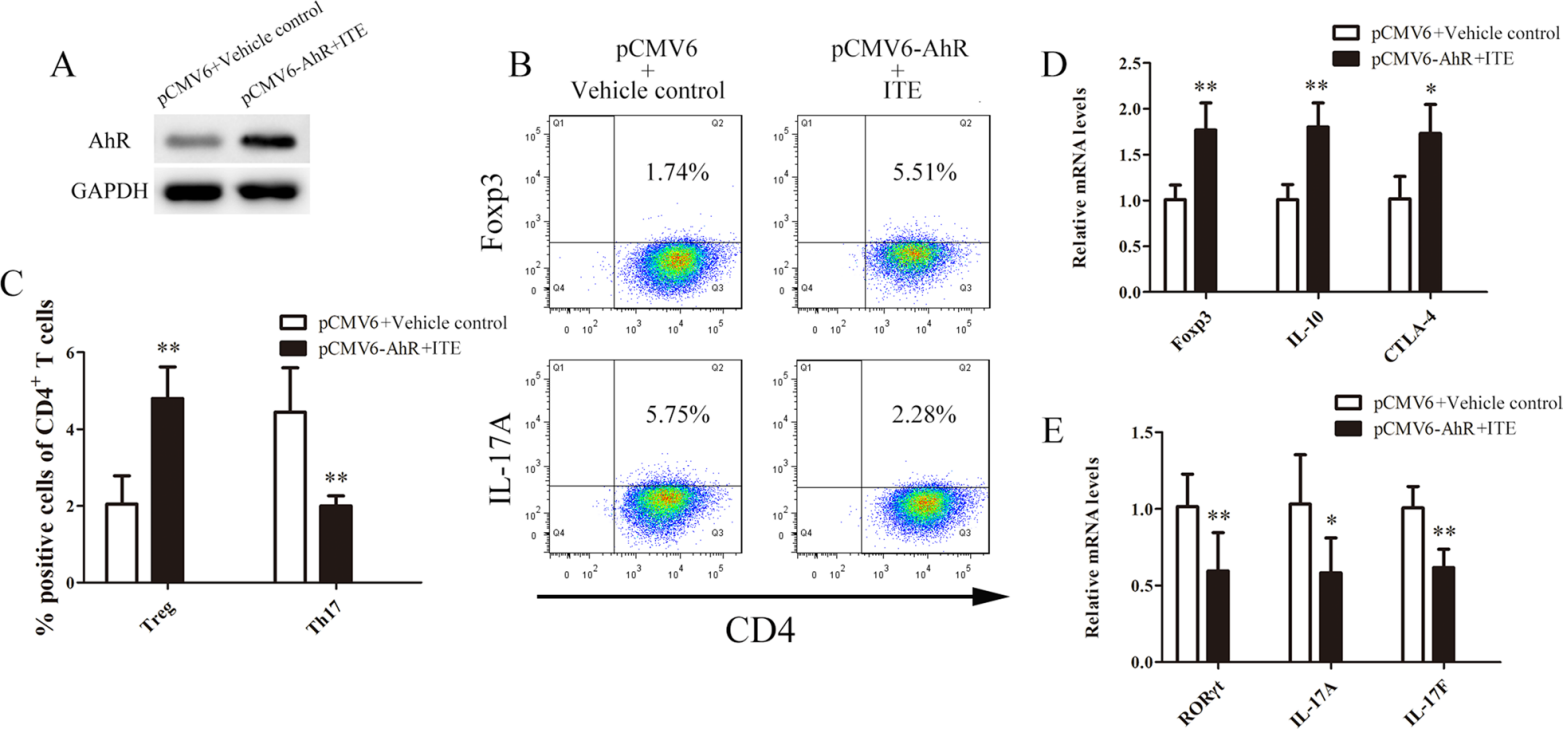
Restoring AhR expression in aGVHD CD4^+^ T cells increases Treg cell amounts and reduces Th17 cell levels. **A** Representative Western blot showing AhR protein levels in aGVHD CD4^+^ T cells transfected with the AhR expression (pCMV6-AhR) or control (pCMV6) plasmid. **B** The percentages of Treg and Th17 cells in the AhR expression plus ITE treatment and transfection control plus vehicle control groups were detected by flow cytometry. **C** Statistical analysis of flow cytometry data. **D**, **E** Expression of genes associated with Tregs (**D**) and Th17 cells **E** assessed by real-time PCR. Data represent the means of three independent experiments (*P < 0.05, **P < 0.01)

To evaluate the effect of restoring AhR expression in CD4^+^ T cells from patients with aGVHD on Tregs and Th17 cells, we transfected the AhR expression plasmid (pCMV6-AhR) or control (pCMV6) plasmid into aGVHD CD4^+^ T cells. Cells in the AhR expression group were treated with ITE, while those transfected with pCMV6 were administered the vehicle control. Flow cytometry showed that the percentage of Tregs in CD4^+^ T cells was significantly higher in the AhR expression plus ITE treatment group compared with the transfection control plus vehicle control group (Figures.2A-C). In contrast, the percentage of Th17 cells was significantly lower in the AhR expression plus ITE treatment group compared with the transfection control plus vehicle control group (Figures.2A-C). Furthermore, we analyzed the expression levels of genes associated with Tregs and Th17 cells by real-time PCR. AhR expression and ITE treatment led to significantly upregulated Treg-associated gene transcripts, including Foxp3, IL-10 and CTLA-4 (Fig. 2D). In contrast, AhR expression and ITE treatment decreased the expression of Th17-associated gene transcripts, including RORγt, IL-17A and IL-17F (Fig. 2E). Collectively, these results demonstrated that restoring AhR expression in aGVHD CD4^+^ T cells could correct Treg/Th17 cell imbalance.

### Insufficient CTCF downregulates the AhR in CD4^+^ T cells from patients with aGVHD

**Fig. 3:**
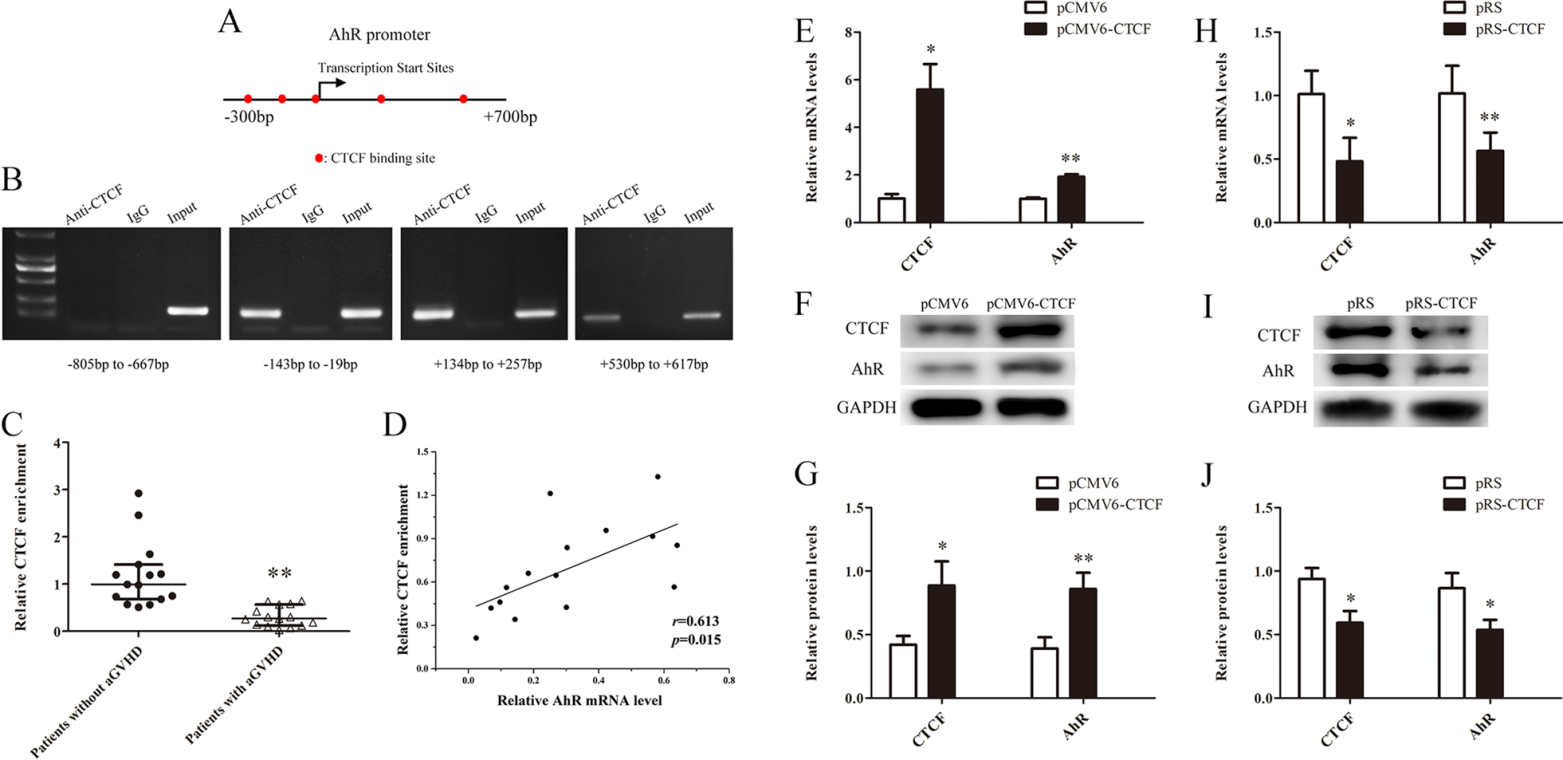
Insufficient CTCF decreases AhR in aGVHD CD4^+^ T cells. **A** Schematic diagram of CTCF binding sites in the AhR promoter region. **B** ChIP-PCR showed that CTCF binds in the AhR promoter region (− 204 bp to + 611 bp). **C** ChIP-qPCR analysis of CTCF enrichment in the AhR promoter in chromatin fractions extracted from CD4^+^ T cells from patients with aGVHD (*n* = 15) and the non-aGVHD group (*n* = 15). Data are relative to input DNA prepared from untreated chromatin. **D** Correlation between CTCF enrichment and AhR mRNA levels in aGVHD CD4^+^ T cells (*n* = 15). **E**–**G** CTCF and AhR expression levels in aGVHD CD4^+^ T cells after CTCF expression (*n* = 3). **E** Relative mRNA levels. **F** Representative Western blot results. **G** Quantitative analysis of band intensities. Data represent the means of three independent experiments. **H**–**J** CTCF and AhR expression levels in normal CD4^+^ T cells after CTCF silencing (*n* = 3). **H** Relative mRNA levels. **I** Representative Western blot results. **J** Quantitative analysis of band intensities. Data represent the means of three independent experiments (*P < 0.05, **P < 0.01)

To explore the molecular mechanism of AhR downregulation in CD4^+^ T cells from aGVHD patients, we analyzed the AhR promoter with an online software (http://jaspar.binf.ku.dk/) and found that the − 204 bp to + 611 bp region contained multiple CTCF binding sites (Fig. 3A). ChIP-PCR analysis was used to assess whether CTCF could interact with the AhR promoter in CTCF over-expressed Jurkat cells. Four pairs of real-time PCR primers covering the AhR promoter (− 805 to + 617 bp) region were used for detection. As shown in Fig. 3B, ChIP-PCR confirmed that CTCF binds in the − 204 to + 611 bp region of the AhR promoter.

Our previous study confirmed that CTCF expression is reduced in CD4^+^ T cells from aGVHD patients compared with non-aGVHD cases [38]. To further explore the binding levels of CTCF in the AhR promoter region in CD4^+^ T cells from aGVHD patients, we assessed the binding levels of CTCF in the AhR promoter region in CD4^+^ T cells from aGVHD and non-aGVHD patients by ChIP-qPCR using anti-CTCF antibodies. As shown in Fig. 3C, aGVHD patients showed reduced CTCF binding to the AhR promoter compared with the non-aGVHD cases. Correlation analysis indicated an obvious positive correlation between CTCF binding and AhR expression in CD4^+^ T cells from aGVHD patients (Fig. 3D).

To further confirm the promoting effect of CTCF on AhR expression, we transfected the CTCF expression (pCMV6-CTCF) or control (pCMV6) plasmid into aGVHD CD4^+^ T cells and transfected the CTCF interference (pRS-CTCF) or negative control (pRS) plasmid into normal CD4^+^ T cells. Real-time PCR and Western blot showed that AhR was significantly upregulated in aGVHD CD4^+^ T cells after CTCF expression (Figures.3E-G), and AhR was significantly downregulated in normal CD4^+^ T cells after CTCF silencing (Figures.3H-J). The above findings suggested that CTCF is an important transcription factor inducing AhR expression, and decreased expression of CTCF might be one of the main causes of AhR downregulation in aGVHD CD4^+^ T cells.

### CTCF recruits TET2 and EP300 to interact with the AhR promoter region

**Fig. 4:**
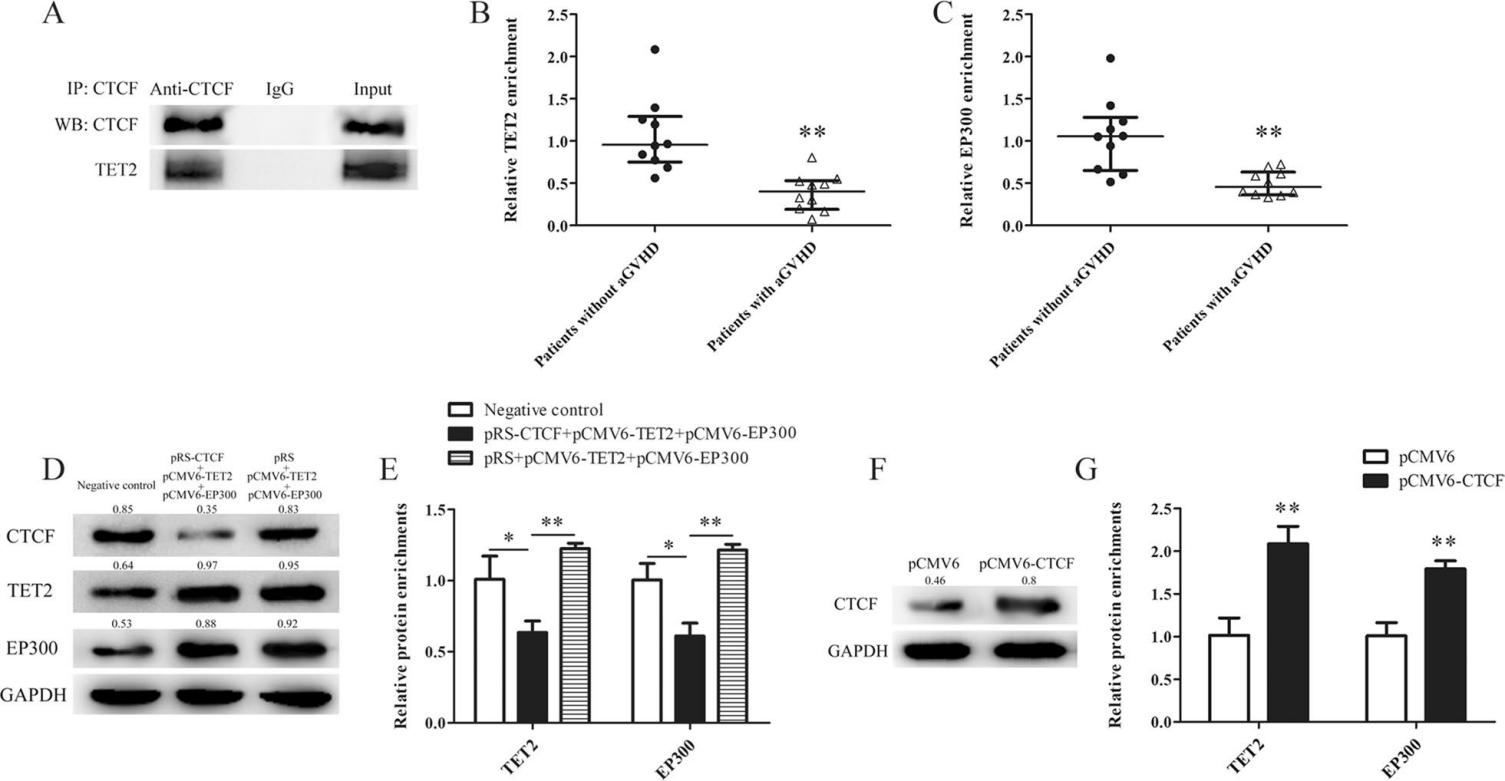
CTCF recruits TET2 and EP300 to interact with the AhR promoter region. **A** Co-immunoprecipitation using anti-CTCF antibodies in Jurkat cells after CTCF and TET2 expression, for the detection of CTCF binding and TET2 by Western blot. **B**, **C** ChIP-qPCR analysis of the enrichment levels of TET2 (**B**) and EP300 **C** in the AhR promoter in chromatin fractions extracted from CD4^+^ T cells from patients with aGVHD (*n* = 10) and the non-aGVHD group (*n* = 10). Results are relative to input DNA prepared from untreated chromatin. **D** Normal CD4^+^ T cells were divided into three groups and transfected with control pRS and pCMV6 plasmids or CTCF silencing (pRS-CTCF) and TET2 and EP300 expression (pCMV6-TET2 and pCMV6-EP300) plasmids, or pRS and pCMV6-TET2 and pCMV6-EP300, respectively. CTCF, TET2 and EP300 protein levels were detected by Western blot. **E** ChIP-qPCR analysis of TET2 and EP300 enrichment levels in the AhR promoter. Results are relative to input DNA prepared from untreated chromatin. Data represent the means of three independent experiments. **F** aGVHD CD4^+^ T cells were divided into two groups and transfected with control pCMV6 and CTCF expression (pCMV6-CTCF) plasmids. CTCF protein levels were detected by Western blot. **G** ChIP-qPCR analysis of TET2 and EP300 enrichment levels in the AhR promoter. Results are relative to input DNA prepared from untreated chromatin. Data represent the means of three independent experiments (*P < 0.05, **P < 0.01)

To further reveal the molecular mechanism of CTCF promoting AhR expression, the hTFtarget database (http://bioinfo.life.hust.edu.cn/hTFtarget#!/) was used to predict regulatory factors that may co-regulate AhR expression with CTCF. TET2 and EP300, a methyl cytosine dioxygenase and a histone acetylase, respectively, were predicted to interact with CTCF. Our previous study confirmed that CTCF in CD4^+^ T cells could bind to EP300; so here we only assessed CTCF interaction with TET2 [38]. First, CTCF and TET2 expression plasmids were transfected into Jurkat cells, and co-immunoprecipitation was used to detect whether CTCF could form a complex with TET2. As shown in Fig. 4A, co-immunoprecipitation confirmed that TET2 was co-precipitated with CTCF. Further, we detected the binding levels of TET2 and EP300 in the AhR promoter region by ChIP-qPCR in CD4^+^ T cells from patients with aGVHD and non-aGVHD cases. As shown in Figures.4B-C, aGVHD patients showed reduced binding levels of TET2 and EP300 at the AhR promoter compared with controls.

To confirm the important role of CTCF in mediating the binding of TET2 and EP300 to the AhR promoter, the CTCF interference and negative control plasmids, respectively, were co-transfected into normal CD4^+^ T cells transfected with TET2 and EP300 expression plasmids (pCMV6-TET2 and pCMV6-EP300), and ChIP-qPCR was used to assess the binding of TET2 and EP300 to the AhR promoter. In the CTCF interference with TET2 and EP300 expression group, the binding levels of TET2 and EP300 in the AhR promoter region were significantly lower compared with those of the scrambled control TET2 and EP300 expression and negative control groups (Figures.4D-E). In addition, we transfected the CTCF expression (pCMV6-CTCF) or control (pCMV6) plasmid into aGVHD CD4^+^ T cells. As shown in Figures.4F-G, TET2 and EP300 binding levels in the AhR promoter region were obviously increased in aGVHD CD4^+^ T cells after CTCF expression. These results suggested that CTCF is an important transcription factor promoting the binding of TET2 and EP300 to the AhR promoter in CD4^+^ T cells. The reduced binding of TET2 and EP300 in the AhR promoter is associated with insufficient expression of CTCF in aGVHD CD4^+^ T cells.

### DNA hypermethylation and histone H3K9/14 hypoacetylation of the AhR promoter in CD4^+^ T cells from patients with aGVHD

**Fig. 5:**
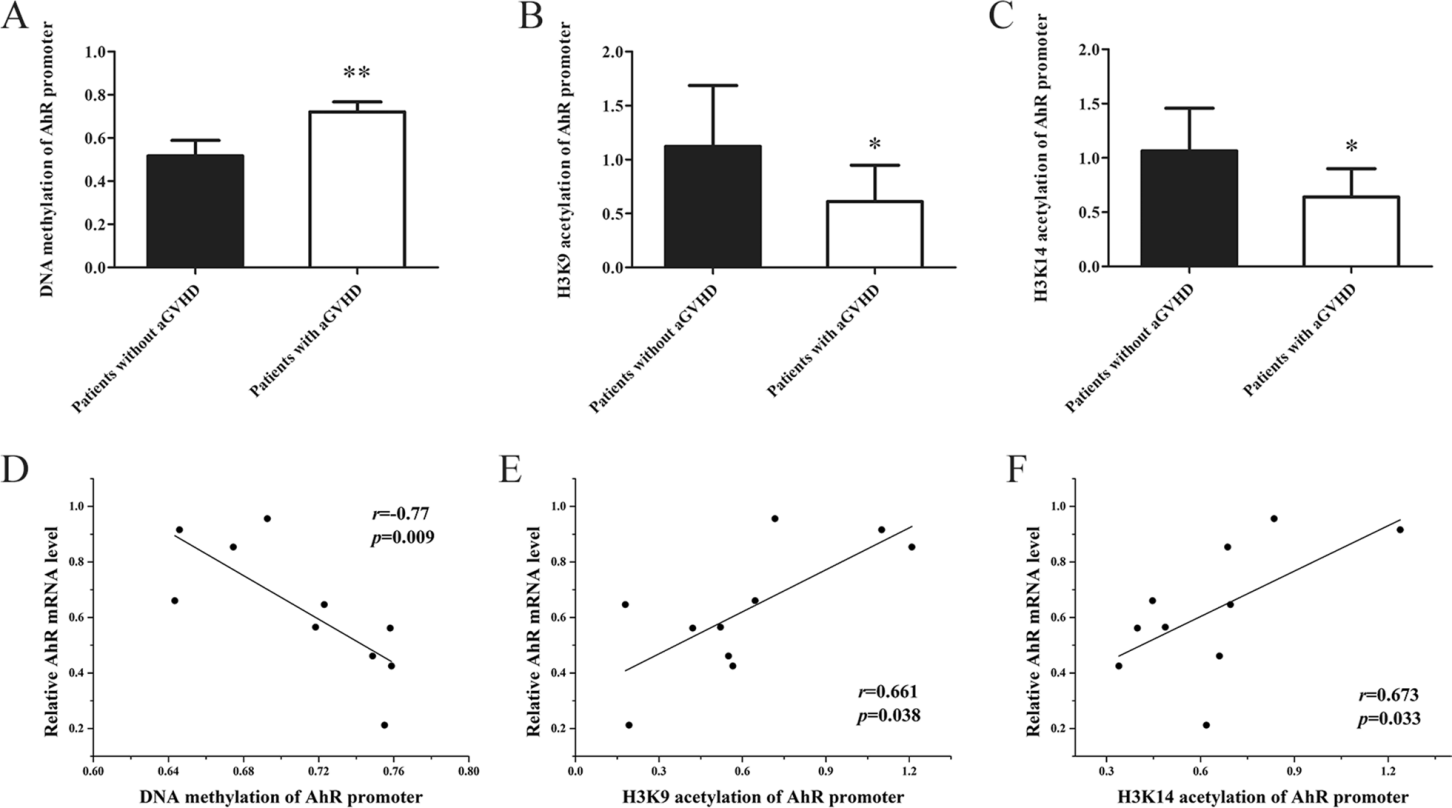
DNA hypermethylation and histone H3K9/14 hypoacetylation of the AhR promoter in aGVHD CD4^+^ T cells. **A** DNA methylation levels of the AhR promoter in CD4^+^ T cells from patients with aGVHD (*n* = 10) and the non-aGVHD group (*n* = 10). **B**, **C** ChIP-qPCR analysis of histone H3K9 (B) and H3K14 **C** acetylation levels in the AhR promoter in CD4^+^ T cells of patients with aGVHD (*n* = 10) and the non-aGVHD group (*n* = 10). Results are relative to input DNA prepared from untreated chromatin. **D**, **F** Correlation analysis of DNA methylation (**D**), H3K9 (**E**)/H3K14 **F** acetylation and AhR mRNA levels. (*P < 0.05, **P < 0.01)

TET2 and EP300 are key enzymes that mediate DNA demethylation and histone H3K9/K14 acetylation, respectively. To confirm whether decreased binding of TET2 and EP300 in the AhR promoter causes DNA hypermethylation and histone hypoacetylation in aGVHD CD4^+^ T cells, we detected DNA methylation and histone H3K9/K14 acetylation levels in the AhR promoter region of CD4^+^ T cells from patients with or without aGVHD. BSP showed that the DNA methylation levels of the AhR promoter in CD4^+^ T cells from patients with aGVHD were significantly higher than those of CD4^+^ T cells from non-aGVHD cases (Fig. 5A). ChIP-qPCR showed that the acetylation levels of histone H3K9 and K14 in the AhR promoter in aGVHD CD4^+^ T cells were significantly lower than those of controls (Figures.5B-C). In addition, DNA methylation and histone H3K9/K14 acetylation levels in the AhR promoter region were correlated with AhR expression. The results showed that DNA methylation was negatively correlated with AhR expression (Fig. 5D), while H3K9/K14ac were positively correlated with AhR expression in aGVHD CD4^+^ T cells (Figures.5E-F).

**Fig. 6:**
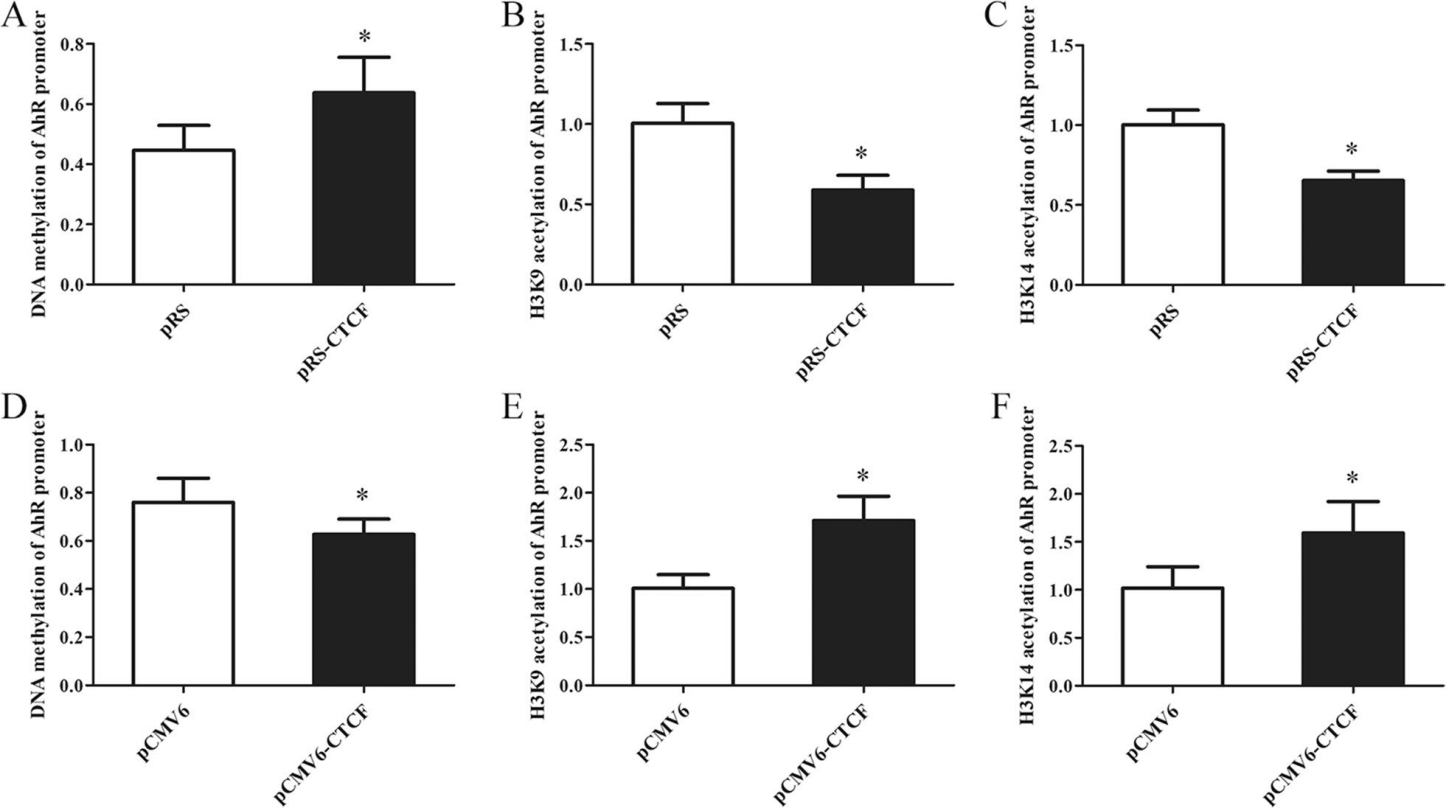
DNA hypermethylation and histone H3K9/14 hypoacetylation of the AhR promoter in CD4^+^ T cells transfected with the CTCF interference or CTCF expression plasmid. **A**–**C** DNA methylation (**A**) and histone H3K9 (**B**)/H3K14 **C** acetylation levels in the AhR promoter region in normal CD4^+^ T cells transfected with pRS or pRS-CTCF. Data represent the means of three independent experiments. **D**–**F** DNA methylation (**D**) and histone H3K9 (**E**)/H3K14 **F** acetylation levels in the AhR promoter of aGVHD CD4^+^ T cells transfected with pCMV6 or pCMV6-CTCF. Data represent the means of three independent experiments (*P < 0.05)

To further confirm the effect of CTCF on DNA methylation and histone H3K9/H3K14 acetylation in the AhR promoter region, DNA methylation and histone H3K9/H3K14 acetylation levels in the AhR promoter region were detected in normal CD4^+^ T cells transfected with the CTCF interference plasmid, or in aGVHD CD4^+^ T cells transfected with the CTCF expression plasmid. We observed increased DNA methylation and decreased histone H3K9/K14ac in the AhR promoter region in normal CD4^+^ T cells after CTCF silencing (Figures.6A-C). Correspondingly, DNA methylation was reduced and H3K9 and K14ac were upregulated significantly in aGVHD CD4^+^ T cells after CTCF expression (Figures.6D-F). Taken together, these results indicated that CTCF deficiency is an important factor in DNA hypermethylation and histone H3K9/K14 hypoacetylation of the AhR promoter in aGVHD CD4^+^ T cells.

## Discussion

CD4^+^ T cells are critical immune effector cells, and alteration in their function has grave consequences on inflammatory responses and immune tolerance [39]. More recently, increasing experimental and epidemiology studies have shown that the AhR plays an important role in immune functions, particularly in the realm of CD4^+^ T cell-mediated inflammation [17, 21].

AhR activation by TCDD promotes the differentiation of Foxp3^+^ Tregs in vivo and in vitro [21, 27]. IL-10-secreting T regulatory type 1 (Tr1) cells are an important subset of CD4^+^ T cells that control excessive inflammation and autoimmunity by inhibiting the functions of antigen-presenting cells and antigen-specific effector T cells [40]. TCDD-mediated AhR activation in CD4^+^ T cells directly changes the expression of genes such as IL-10, CTLA4, CD39 and GITR, and promotes CD4^+^ T cell differentiation into Tr1 cells [41–44]. In contrast to an increase in Tregs by TCDD, another high-affinity AhR ligand, FICZ, has been reported to enhance Th17 responses and exacerbate immune-mediated diseases in several mouse models [21, 45]. These seemingly contradictory results may be related to the activation strength of the AhR signal. New studies find that low doses of AhR ligands (TCDD, FICZ) did not induce Tregs nor alter the alloresponse, but instead increased the percentage of CD4^+^ T cells that produce IL-17. When administered at high doses, TCDD and FICZ induced Tr1 cells and increased Foxp3^+^ Tregs in conjunction with suppression of the alloresponse [46]. These data indicate that AhR has an impact on both Treg and Th17 differentiation, but Tregs may have a greater demand for activation intensity of AhR signal. Th17 is more sensitive to AhR signal, and a small amount of AhR activation can maintain the differentiation of Th17. We speculated that the attenuation of AhR signal in CD4^+^ T cells has a greater impact on the differentiation of Tregs, resulting in the dominance of Th17 cells.

In this study, AhR expression was significantly downregulated in CD4^+^ T cells from patients with aGVHD compared with those from non-aGVHD cases. In addition, our results confirmed that after activating AhR deficient CD4^+^ T cells, the expression levels of the activation markers-CD40L, CD134 and CD137 and cell proliferation activity were significantly higher than those of AhR-expressing CD4^+^ T cells. Studies have shown that AhR endogenous ligands in the plasma of aGVHD patients are significantly reduced, indicating that the AhR signaling pathway is inhibited in aGVHD patients [47, 48]. These results suggest that the normal expression and activation of AhR are important factors in preventing CD4^+^ T cell over activation and proliferation, which may be related to AhR promoting Treg cell differentiation and function. Restoring the expression of AhR in aGVHD CD4^+^ T cells and fully activating it can promote the differentiation of Tregs and induce the expression of functional genes, inhibiting the activation and proliferation of CD4^+^ T cells.

CTCF is a ubiquitously expressed and multifunctional transcription factor implicated in many key cellular processes, including promoter activation and repression, hormone-responsive gene silencing, and genomic imprinting. It is essential and highly conserved from Drosophila to mice and humans [49, 50]. Of note, it was recently shown that CTCF may be closely related to Th2 cell and Treg differentiation. Th2 polarization of CTCF-deficient CD4^+^ T cells was blocked, and the transcription levels of IL-4, IL-5 and IL-13 were strongly reduced. In addition, the differentiation of CTCF-deficient CD4^+^ T cells into Tregs was also suppressed, and Foxp3 expression was decreased [51]. CTCF cooperates with Oct-1 to directly interact with the IL-17 locus, regulate Th17 cell differentiation and IL-17 production [52]. These studies showed that CTCF is involved in increasing Tregs and reducing Th17 cells.

Our previous studies have confirmed that CTCF interacts with EP300 to directly bind to the p53 promoter region and promotes p53 expression by upregulating histone H3K9/K14 acetylation levels in this region. The low expression of CTCF in CD4^+^ T cells from aGVHD cases is an important factor leading to histone H3K9/K14 hypoacetylation in the p53 promoter and downregulation p53. Restoring CTCF expression in CD4^+^ T cells from aGVHD patients increases p53 amounts and corrects the imbalance of Th17 cells/Tregs [38]. In the present study, we confirmed that CTCF is an important activator of AhR transcription. It recruited EP300 and TET2 to bind to the AhR promoter region and promoted AhR expression by mediating histone H3K9/K14 hyperacetylation and DNA demethylation in this region. The low expression of CTCF caused histone hypoacetylation and DNA hypermethylation of the AhR promoter, resulting in insufficient expression in aGVHD CD4^+^ T cells.

EP300 is one of several acetyltransferases that regulate Foxp3 acetylation and functions. Conditional deletion or pharmacologic inhibition of EP300 in Foxp3^+^ Tregs increases T cell receptor-induced apoptosis in Tregs and impairs Tregs’ suppressive function as well as peripheral Treg cell induction [53, 54]. TET2 is a key factor that catalyzes the demethylation of the Treg-specific demethylated region (TSDR) of the Foxp3 gene. TET2 mediates TSDR demethylation and activates Foxp3 transcription, driving regulatory T cell differentiation. TET2 deletion leads to Foxp3 hypermethylation, impairs Treg cell differentiation and function, as well as autoimmune disease [55, 56]. Thus, EP300 and TET2 promote the differentiation and functional maintenance of Treg cells by upregulating Foxp3. The current findings provide new insights into the molecular mechanism of EP300 and TET2 regulating the balance of Treg/Th17 cell ratio and participating in immunosuppression.

## Conclusions

The present data emphasize the critical role of CTCF in the maintenance of stable AhR expression. Insufficient CTCF expression leads to excessively low expression of AhR, resulting in excessive activation of CD4^+^ T cells and Th17 cell/Treg ratio imbalance, thereby mediating the occurrence of aGVHD.

## Materials and methods

### Subjects

Totally 55 patients administered allo-HSCT from HLA-identical sibling donors in the Central of Hematopoietic Stem Cell Transplantation of Xiangya Hospital from 2017 to 2021 were included. All subjects provided signed informed consent in accordance with the Declaration of Helsinki. This study was carried out in accordance with the recommendations of international ethical guidelines for biomedical research involving human subjects. The study protocol was approved by the Human Ethics Committee of Xiangya School of Medicine, Central South University. The clinical characteristics of the included patients are shown in Table 1. Conditioning regimens were based on our previous study [57]. aGVHD was assessed based on clinical symptoms in accordance with commonly accepted criteria [58, 59]. We analyzed samples from patients at onset of aGVHD (*n* = 55) and time-matched samples from patients without aGVHD (*n* = 55). Peripheral blood samples were collected as soon as possible after aGVHD diagnosis and before starting the therapy. In addition, we collected normal CD4^+^ T cells from 12 medical professionals recruited in Xiangya Hospital for transfection.

**Table 1:** Clinical Characteristics of Patients

	No GVHD	aGVHD
Number	55	55
Median age	33	35
Sex (female/male)	25/30	23/32
*Diagnosis*		
ALL	19	15
AML	29	30
MDS	6	8
CML	1	2
*Acute GVHD grade*		
1		8
2		25
3		19
4		3
Days to aGVHD onset, median (range)		51 (range: 22–93)

### Isolation, culture and transfection of CD4^+^ T cells

Peripheral blood mononuclear cells (PBMCs) were obtained by Ficoll density gradient. CD4^+^ T cells were purified from PBMCs using human CD4 beads (Miltenyi, Bergisch Gladbach, Germany) and cultured in RPMI 1640 (Thermo Fisher Scientific, MA, USA) containing 10% FBS, and 1% penicillin/streptomycin. The isolated CD4^+^ T cells were electrotransfected at 2 × 10^6^/sample. Human T cell nucleofector Kit and Amaxa nucleofector (Lonza, MD, USA) were used to transfect the gene expression (pCMV6) and interference (pRS) plasmids into CD4^+^ T cells. Briefly, CD4^+^ T cells were mixed with the plasmid in 100 μl of human T cell nucleofector solution. The mixture was electrotransfected using nucleofector program V-024 in the Amaxa nucleofector. The transfected cells were cultured in RPMI 1640 complete medium for 48 h.

### ITE treatment, activation and proliferation of CD4^+^ T cells

(1′H-indole-3′-carbonyl)-thiazole-4-carboxylic acid methyl ester (ITE) (MedChemExpress, NJ, USA) was dissolved in DMSO. CD4^+^ T cells from healthy donors were transfected with pRS-AhR and pRS plasmids, respectively, and stimulated with ITE (40 μM) [60]. Then, cells were activated with Dynabeads T cell expander (anti-CD3/CD28; Invitrogen, CA, USA) for 3 days. The expression levels of T cell activation markers, including CD40L, CD134 and CD137, were detected by flow cytometry. In addition, CD4^+^ T cells were transfected with pRS-AhR and pRS plasmids, respectively, and treated with ITE; cell proliferation was stimulated with Dynabeads T cell expander plus IL-2. After 4 days, the cells were inoculated in 96-well plates. Then, 10 µL of Cell Counting Kit-8 (CCK-8, MedChemExpress) was added to each well, and the plate was incubated at 37 °C for 1 h in a 5% CO_2_ incubator. Absorbance was measured at 450 nm on a microplate reader.

After aGVHD CD4^+^ T cell transfection with AhR expression (pCMV6-AhR) or control (pCMV6) plasmid, cells overexpressing AhR were treated with ITE, while control cells were administered the vehicle control (DMSO). Simultaneously, all transfected cells were stimulated by Dynabeads T cell expander for 3 days. The percentages of Treg and Th17 cells in each group were detected by flow cytometry. The expression levels of genes associated with Treg (Foxp3, IL-10 and CTLA-4) and Th17 (RORγt, IL-17A and IL-17F) cells were detected by real-time PCR.

### Flow cytometry and intracellular cytokine staining

Flow cytometry was performed to assess the expression levels of the T cell activation markers CD40L, CD134 and CD137. Briefly, the activated cells were suspended in ice-cold FACS buffer (PBS-2% FBS) and incubated with fluorochrome labeled mAbs, including anti-CD40L-PE (eBiosciences, Thermo Fisher, 24–31), anti-CD134-FITC (eBiosciences, ACT-35) and anti-CD137-APC (eBiosciences, 4-1BB) for 20 min at 4 °C. As a control, cells were stained with isotype-control mAbs. Cells were then washed in ice-cold FACS buffer and analyzed on a BD FACScan with the Cell Quest software (BD Biosciences, NJ, USA).

Tregs were detected with anti-CD4-PerCP (eBiosciences, MEM-241) and anti-Foxp3-PE mAbs (eBiosciences, PCH101), while Th17 cells were detected with anti-CD4-PerCP and anti-IL-17A-FITC mAbs (eBiosciences, eBio64DEC17). Briefly, for Tregs, activated cells were first surface-stained with anti-CD4-PerCP mAb. Then, the cells were fixed and permeabilized for intracellular staining with the 4% paraformaldehyde fix solution and Perm/Wash buffer (BD Biosciences, NJ, USA), respectively. After fixation and permeabilization, they were stained with anti-Foxp3-PE and analyzed on a BD FACScan. Meanwhile, Th17 cells were incubated for 4 h with 50 nM PMA (Sigma Aldrich) and 0.5 μg/ml ionomycin (Invitrogen, Thermo Fisher) in the presence of 5 g/mL brefeldin A (eBiosciences) in a tissue culture incubator at 37 °C and 5% CO_2_. Cells were then labeled with anti-CD4-PerCP mAbs, fixed and permeabilized, and stained with anti-IL-17A-FITC mAbs. As a control, cells were also stained with isotype-control mAbs.

### RNA isolation and real-time PCR

The TRIzol Reagent (Invitrogen, CA, USA) was used to isolate total RNA from CD4^+^ T cells. After DNase treatment, RNA was reverse transcribed with SuperScript II reverse transcriptase (Invitrogen), and then, cDNA was amplified by real-time PCR with fluorescent dye SYBR Green Master Mix (Thermo Fisher Scientific, MA, USA) using an ABI Prism 7500 (Thermo Fisher Scientific). The housekeeping gene human glyceraldehyde-3-phosphate dehydrogenase (GAPDH) was used as internal standard for sample normalization. Data analysis was performed by the 2^−ΔΔCt^ method; ΔΔCt = (Ct_target gene_ − Ct_internal control_)_sample_ − (Ct_target gene_ − Ct_internal control_)_control_. The primers are listed in Table 2.

**Table 2:** Primer sequences for real-time qPCR

	Forward primer	Reverse primer
AhR	TCCACTTCAGCCACCATC	GGGACTCGGCACAATAAA
CTCF	GAGGCTGCTGTGGACGAT	CAGGCAAAGGTAGGGTGTG
Foxp3	GAGAAGCTGAGTGCCATGCA	AGAGCCCTTGTCGGATGAT
IL10	TGAGAACAGCTGCACCCACTT	TCGGAGATCTCGAAGCATGTTA
CTLA-4	CAGACCTGCCATGCCAATTTG	GGCCCATATACTTGGAATGGAACA
RORγt	GCTGGTTAGGATGTGCCG	GGATGCTTTGGCGATGA
IL-17A	CAATCCCACGAAATCCAGGATG	GGTGGAGATTCCAAGGTGAGG
IL-17F	TGCTCAAGGAAAGGAAGACA	ATGGTGGATGACAGGGGTG
GAPDH	AAGAGCTACGAGCTGCCTGAC	ATGGCCCAGCGGATGAG

### Western blot

CD4^+^ T cells were lysed in ice-cold lysis buffer containing proteinase inhibitors (Thermo Fisher Scientific). Then, the lysate was centrifugated at 12,000 g for 15 min at 4˚C. Protein concentrations were determined with the Bradford protein assay kit (Bio-Rad, CA, USA). Next, equal amounts of protein were resolved by 8% sodium dodecyl sulfate polyacrylamide gel electrophoresis (SDS-PAGE), followed by electro-transfer onto polyvinylidene difluoride (PVDF) membranes (Bio-Rad, CA, USA). After blocking at 37 °C with 5% milk in PBS containing 0.1% Tween-20 for 2 h, the membranes were incubated with primary antibodies overnight at 4 °C. The primary antibodies tested included anti-AhR, anti-CTCF, anti-TET2, anti-EP300 and anti-GAPDH (Cell Signaling Technology, MA, USA). After extensive washing, the membranes were incubated with horseradish peroxidase–conjugated secondary antibodies for 2 h at room temperature. The Quantity One software (Bio-Rad, CA, USA) was used to assess immunoreactive bands.

### Chromatin immunoprecipitation (ChIP)

ChIP analysis was performed with SimpleChIP® Plus Sonication Chromatin IP Kit (Cell Signaling Technology) according to the manufacturer’s instructions. Briefly, CD4^+^ T cells were fixed for 10 min with 1% formaldehyde at room temperature. Then, cross-linking was stopped with glycine at 0.125 M. The cells were washed twice with ice-cold PBS and then lysed. Chromatin was sheared into 500–1000 base pair fragments. Next, anti-CTCF, anti-TET2, anti-EP300, anti-H3K9ac, anti-H3K14ac and control rabbit IgG (Cell Signaling Technology), respectively, were added to the lysates for overnight incubation. Immune complexes were precipitated with protein A agarose beads, washed and eluted with 100 μl of TE buffer containing 0.5% SDS and 200 μg/ml proteinase K. Purified DNA was used to amplify the target fragment by PCR or real-time PCR. Primers used were as follows: Forward 1 (− 805 to − 787):5′ CCCTTCACTCCCCCTACA 3′ and Reverse 1 (− 685 to − 667):5′TGGGCCTGCAAATAACAT3′, Forward 2 (− 143 to − 121):5′ GCCCTCAAGGAAGACGGAATG3′ and Reverse 2 (− 40 to − 19):5′ ACCGGCTGAATAGCAGGAGCA3′, Forward 3 (+ 134 to + 155):5′ GCGGGAAGCACCCTGGATTTA′ and Reverse 3 (+ 236 to + 257):5′ TAGAATCCTGGCCTGGGTCGC′, Forward 4 (+ 530 to + 552):5′ CCGCAGTGGTCCCAGCCTACAC′ and Reverse 4 (+ 598 to + 617):5′ TCATGGTGCCCAGCCGACG′.

### Co-Immunoprecipitation

Nuclear proteins from CD4^+^ T cells were extracted with NE-PER™ Nuclear and Cytoplasmic Extraction Reagents (Thermo Fisher Scientific). Then, anti-CTCF antibodies and control rabbit IgG, respectively, were added to nuclear extracts for overnight incubation at 4 °C. Protein A/G PLUS-agarose beads (Millipore) were added to each IP reaction for 2 h with rotation at 4˚C. Agarose beads were harvested by centrifugation at 3000 g for 2 min. The bound proteins were eluted from the solid support with SDS sample loading buffer. These complexes were analyzed by Western blot using anti-CTCF and anti-TET2 primary antibodies.

### Genomic DNA extraction and bisulfite sequencing

Genomic DNA was isolated from CD4^+^ T cells with TIANamp Genomic DNA Kit (TIANGEN, Beijing, China). Bisulfite conversion was performed with EpiTect Bisulfite Kit (Qiagen, CA, USA). Two CpG islands within the AhR promoter region were amplified by PCR. Then, PCR products were subcloned into the pGEM-T vector (Promega, WI, USA). Eight independent clones were sequenced for each of the amplified fragments. Primers used were as follows:5′ –GGTGGGGTTTTTAAGGAAGA-3′(forward1) and 5′ –AAACTCCCCAACCACTACCTC-3′(reverse1); 5′ –GGTATTTTTTTTATTTAAGGGGT-3′(forward2) and 5′ –TACAAATAAACTACCTAAACCTAAC-3′(reverse2);

### Statistical analysis

Statistics were performed using SPSS 22.0 software. Continuous variables with normal distribution were expressed as mean ± SD; non-normally distributed variables were reported as medians with interquartile ranges. Normally distributed continuous variables were compared by independent samples *t* test. Non-normally distributed variables were compared by Mann–Whitney *U* test. Correlations were analyzed using Spearman’s correlation coefficients. Statistical significance was defined by *P* < 0.05.

## Data Availability

The datasets supporting the conclusions of this article are included within the article. All other datasets used and analyzed during the study are available from the corresponding author on reasonable request.
